# Gut Microbiota Diversity of Local Egyptian Cattle Managed in Different Ecosystems

**DOI:** 10.3390/ani14182752

**Published:** 2024-09-23

**Authors:** Hadeer M. Aboshady, Asimenia Gavriilidou, Nasser Ghanem, Mohamed A. Radwan, Ahmed Elnahas, Rania Agamy, Nadia H. Fahim, Mohamed H. Elsawy, Al-Moataz Bellah M. Shaarawy, Ahmed M. Abdel-Hafeez, Juha Kantanen, Catarina Ginja, Mahlako L. Makgahlela, Donald R. Kugonza, Rayner Gonzalez-Prendes, Richard P. M. A. Crooijmans

**Affiliations:** 1 Department of Animal Production, Faculty of Agriculture, Cairo University, Giza 12613, Egyptm.radwan883@agr.cu.edu.eg (M.A.R.); rania.agamy@agr.cu.edu.eg (R.A.); nadiaamn@agr.cu.edu.eg (N.H.F.); 2 Laboratory of Microbiology, Wageningen University & Research, 6708 WE Wageningen, The Netherlands; asimenia.gavriilidou@wur.nl; 3 Animal Production Department, Faculty of Agriculture, Sohag University, Sohag 82524, Egypt; ahmed.abdelrahman@agr.sohag.edu.eg; 4 Department of Cattle, Animal Production Research Institute, Agriculture Research Center, Dokki, Giza 12618, Egypt; mhaesawy@yahoo.com (M.H.E.); am.shaarawy@arc.sci.eg (A.-M.B.M.S.); dr_hafeez2008@yahoo.com (A.M.A.-H.); 5 Natural Resources Institute Finland, 31600 Jokioinen, Finland; juha.kantanen@luke.fi; 6 CIISA, Faculty of Veterinary Medicine, University of Lisbon, 1300-477 Lisboa, Portugal; 7 CIBIO, Research Centre in Biodiversity and Genetic Resources, InBIO, Associate Laboratory, BIOPOLIS Program in Genomics, Biodiversity and Land Planning, University of Porto, 4485-661 Vairão, Portugal; 8 Agricultural Research Council, Animal Production, Private Bag X2, Irene 0062, South Africa; mmakgahlela@arc.agric.za; 9 Department of Animal, Wildlife and Grassland Sciences, University of the Free State, P.O. Box 339, Bloemfontein 9301, South Africa; 10 School of Agricultural Sciences, College of Agricultural and Environmental Sciences, Makerere University, Kampala P.O. Box 7062, Uganda; 11 Animal Breeding and Genomics, Wageningen University & Research, 6700 AH Wageningen, The Netherlands; rayner.prendes@gmail.com (R.G.-P.); richard.crooijmans@wur.nl (R.P.M.A.C.)

**Keywords:** local breed, 16S rRNA sequencing, prokaryotes, biodiversity

## Abstract

**Simple Summary:**

The gut microbiota has provided valuable insights into understanding an animal’s adaptation to its environment. Additionally, it has a significant effect on the animal’s performance. Comprehending the composition of the microbiota and its interaction with its host is essential for formulating knowledge-based strategies aimed at improving animal adaptability and productivity. This study aimed to investigate the diversity of the microbiota of local Egyptian cattle in three different ecosystems to gain insights into the potentiality of the adaptation of local Egyptian cattle’s microbiota. The results suggest an adaptive response of the animals to their respective/specific environments, with a clear effect of both heat stress and feed type. These findings could be useful in enhancing animal adaptations and productivity.

**Abstract:**

The animal gastrointestinal tract contains a complex microbiome whose composition ultimately reflects the co-evolution of microorganisms with their animal host and their host’s environment. This study aimed to gain insights into the adaptation of the microbiota of local Egyptian cattle to three different ecosystems (Upper Egypt, Middle Egypt, and Lower Egypt) distributed across 11 governorates (with an average of 12 animals per governorate) using amplicon sequencing. We analyzed the microbiota from 136 fecal samples of local Egyptian cattle through a 16S rRNA gene sequencing approach to better understand the fecal microbial diversity of this breed which developed under different ecosystems. An alpha diversity analysis showed that the fecal microbiota of the Egyptian cattle was not significantly diverse across areas, seasons, sexes, or farm types. Meanwhile, microbiota data revealed significant differences in richness among age groups (*p* = 0.0018). The microbial community differed significantly in the distribution of its relative abundance rather than in richness across different ecosystems. The taxonomic analysis of the reads identified *Firmicutes* and *Actinobacteriota* as the dominant phyla, accounting for over 93% of the total bacterial community in Egyptian cattle. Middle Egypt exhibited a different microbial community composition compared to Upper and Lower Egypt, with a significantly higher abundance of *Firmicutes* and *Euryarchaeota* and a lower abundance of *Actinobacteriota* in this region than the other two ecosystems. Additionally, Middle Egypt had a significantly higher relative abundance of the *Methanobacteriaceae* family and the *Methanobrevibacter* genera than Lower and Upper Egypt. These results suggest a difference in the adaptation of the fecal microbial communities of Egyptian cattle raised in Middle Egypt. At the genus level, eleven genera were significantly different among the three ecosystems including *Bacillus*, *DNF00809*, *Kandleria*, *Lachnospiraceae_NK3A20_group*, *Methanobrevibacter*, *Mogibacterium*, *Olsenella*, *Paeniclostridium*, *Romboutsia*, *Turicibacter*, and *UCG-005*. These significant differences in microbiota composition may impact the animal’s adaptation to varied environments.

## 1. Introduction

In the Near East and North Africa, livestock production is crucial to national economies and food security [1]. Small-scale farmers keep the majority of Egypt’s livestock, with government farms accounting for less than 2% of the total livestock population [2]. Egypt’s cattle population was estimated to be 5.01 million heads in 2016 [3]. Climate change poses one of the greatest threats to animal productivity in tropical and subtropical regions, leading to reduced growth, reproduction, and milk production and dramatic changes in physiological functions due to heat stress [4]. Besides the risk of heat stress, climate changes have impacted pathogen susceptibility and gut microbial communities, which subsequently impact cattle diseases’ spread and their productivity [5]. The adaptation of farm animals is therefore necessary to enable an adequate response to climate change. In addition, this physiological trait is important for sustainable food security, livelihood demands, and natural resource conservation. One of the best strategies for coping with these adverse climatic conditions is the genetic selection of resistant/tolerant individuals [6]. Indeed, the genetic adaptability of local breeds makes them well fit to their environments, highlighting their importance as an integral part of the rural lifestyle.

Local cattle raised in Egypt are exposed to various ecosystems/stressors, ranging from Mediterranean weather in the north (Lower Egypt) to very hot and dry conditions in the south (Upper Egypt), with an expectation of more severe heat stress in Upper than Lower Egypt in the coming decades [7]. Interestingly, it has been shown that the environment affects the composition of the rumen’s microbiota, which directly influences animal productivity [8]. Recent studies have indicated that gut microbiota composition has the potential to adapt to different environments that are affected by geography [9] and intense heat stress [10,11]. Recently, there has been increasing focus on the importance of microbiota diversity as a key regulator of animal health, adaptation, and production [12]. Another important focus of microbiota research is to reduce methanogens in order to decrease greenhouse gas emissions from ruminants [13]. Microbiomics is a promising way to examine the alterations in the gut microbial composition that result from animals’ adaptation to different environments [9,10,11]. Although several studies have assessed the differences between fecal and ruminal microbiota, showing that ruminal microbial populations are more diverse than fecal populations [14,15], the fecal microbiota’s profile has still demonstrated a regulatory effect and an association with health and productive traits [16,17]. Additionally, fecal sampling has proven to be a viable, less intrusive technique that is appropriate for establishing a connection between alterations in the quantity (abundance) and variety (diversity) of the gut microbial population, which is subsequently associated with functional traits [18].

This study aimed to gain insights into the adaptation of the microbial community of local Egyptian cattle that were managed across three ecosystems using high-throughput sequencing of the 16S rRNA gene.

## 2. Materials and Methods

### 2.1. Ethical Approval

Animal welfare ethical approval for different biological sample collection protocols was given by the Institutional Animal Care and Use Committee (CU-IACUC), located in Cairo University (Giza, Egypt), under number CUIIF720.

### 2.2. Farming System

Animals were managed under two different systems of animal care and management (farm types). The first system is an intensive system (*n* = 37), with animals distributed across three herds of Egyptian cattle. One herd was raised in Middle Egypt (*n* = 18; Seds Research Station, Beni Suef governorate) and the second was raised in Upper Egypt (*n* = 10; El Serw Research Station, Damietta governorate). Both herds belong to the Animal Production Research Institute (APRI). The third intensive herd of Egyptian cattle was raised on a commercial feedlot farm located in Assiut governorate (*n* = 9; Lower Egypt). The second type of management is an extensive system (*n* = 99), where animals are kept in small herds of 1–5 individuals. Animals in this extensive system are housed in the same building as the farmer, in a separate area, or nearby them.

Despite the presence of a few commercial farms, the extensive management system remains the predominant method for managing Egyptian cattle in Egypt. The number of animals selected from each system in this study was based on the availability of animals under either commercial or extensive management. Additionally, due to the limited number of Egyptian cattle in certain regions of Egypt, this study only included governorates that continue to manage this breed.

The samples were classified based on several factors: sex (male and female), age group (≤2 years, 3–4 years, 5–6 years, 7–8 years, and ≥8 years), geographic region (Lower, Middle, and Upper Egypt), season of the year (hot/summer: April to October, cold/winter: November to March), and farm management system used (intensive vs. extensive). Detailed information on the environmental conditions during sampling is presented in Table 1. These conditions were sourced from the Central Laboratory for Agricultural Climate at the Agricultural Research Center, Egyptian Ministry of Agriculture & Land Reclamation. The temperature–humidity index was calculated following the method outlined by Mader et al. [19]:

THI = (0.8 × Tdb) + [(RH/100) × (Tdb − 14.4)] + 46.4

Temperature–humidity index (THI) = (0.8 × ambient temperature) + [(% relative humidity/100) × (ambient temperature − 14.4)] + 46.4.

### 2.3. Feeding Management

Cows managed under an intensive system were fed berseem (*Alfa alfa*), concentrate according to their requirements and rice straw ad libitum during the hot season. In the cold season, these animals were offered the same, but the berseem was replaced by corn silage. On the other hand, local Egyptian cattle that were reared under an extensive management system were offered berseem ad libitum and a hay wheat and concentrate ration mixture (corn grain and wheat bran) during the hot season. The animals were fed the same ration during the cold season, but the berseem was replaced by green corn fodder ad libitum.

The feeding regime for Egyptian cattle in the intensive system is based on calculated requirements for both maintenance and performance and includes both concentrate and roughage. Their maintenance and performance needs (for milk and meat production) were determined in accordance with the standard guidelines of the Nutrient Requirements of Cattle (NRC) [20].

The housing of Egyptian cows varied depending on their management system. In the intensive system, the animals were housed in open yards shaded by roofs made of either cement or metal sheets. The average dimensions of a yard for 10 animals were 15 m in length and 10 m in width, and the height of the shaded ceiling was 6 m. In contrast, animals managed under the extensive system were typically kept in semi-enclosed housing with variable dimensions and different types of shading. These animals were usually kept in small herds of 1–5 individuals per farm.

### 2.4. Fecal Sample Collection

A total of 136 fecal samples were collected from Egyptian cattle (26 males and 110 females) spread over 11 governorates. The primary criterion for selecting animals was that only Egyptian cattle were chosen, as they are well known for their superior disease resistance compared to foreign breeds like Holstein [21]. Therefore, one of the main aims of this study was to find out the microbiota of this breed. The second key criterion for selecting animals was ensuring that the selected animals had no familial or genetic relationship with each other, in order to eliminate the influence of genetic factors. The third criterion was to select an equal number of males and females. However, due to farmers’ preference for breeding foreign cattle to increase revenue, the number of Egyptian cattle has been decreasing sharply. Additionally, males are primarily used for mating or fattening, which leads to their frequent slaughter, resulting in a lower number of males compared to females in this study. On average, 12 animals per governorate were enrolled in this study. Fecal samples were collected directly from the rectum into 50 mL falcon tubes, cooled to 4 °C, and then transported and stored at −20 °C until DNA extraction.

### 2.5. DNA Extraction and Sequencing of 16S rRNA Gene

DNA was extracted using 120 mg of the fecal sample and the Maxwell 16 Tissue LEV Total RNA Purification Kit (Promega, Madison, WI, USA). The concentration of the DNA samples was determined using the Qubit dsDNA BR assay kit (Invitrogen, Fisher Scientific, Waltham, MA, USA) according to the manufacturer’s instructions. The microbiota composition was analyzed with barcoded amplicons of the V4 region of the 16S rRNA gene generated using the F515-806R primer set [22]. The amplification reactions were performed in triplicate, as described elsewhere [23]. After confirmation of the right size of the amplicons by agarose gel electrophoresis, PCR products were purified with the HighPrep kit (MagBioEurope Ltd., Kent, UK) following the manufacturer’s instructions. PCR products were pooled in equimolar amounts and sequenced on the Illumina NovaSeq 6000 platform (GATC-Biotech, Konstanz, Germany). To control for potential technical biases, two human gut mock synthetic communities [24] and three rumen mock synthetic communities (generated in-house) were included as positive controls and PCR reactions with no DNA template were used as negative controls.

### 2.6. Bioinformatics Analysis

The quality of the samples was assessed using FASTQC (v0.12.1), and amplicon sequencing variants (ASVs) were identified and classified using NGTax 2.0 in combination with the SILVA 138.1 database [25]. In order to avoid the inclusion of spurious ASVs caused by sequencing and PCR errors, a threshold of a 0.1% relative abundance was applied per sample.

For the assessment of the alpha and beta diversities of the bacterial community and the description of their composition, the ASVs were rarefied to equal sample sizes based on the sample with the fewest sequences (4505), to correct for uneven sampling depth, using a random subsampling procedure programmed in the statistical software package R 4.3.0 (R Development Core Team, Vienna, Austria) [26].

Diversity within samples (α-diversity) was calculated using Shannon’s diversity index and the phyloseq package in R software [27]. Beta diversity was calculated by employing the Jaccard index of similarity. The level of significance was determined at *p* < 0.05. Microbial profile data were displayed using the plotbar in the ggplot2 and phyloseq packages in the R software. To visualize the impact on the microbial community of each group of animals, phyla, families, and genera were displayed in plot bars with the phyloseq package. The differences in the proportions of certain phyla, families, and genera were highlighted in the plot bar with their relative abundances. The search for components to describe and discriminate samples according to their diversity and abundance was carried out using a Partial Least Squares Discriminant (PLSD) analysis in mixomics software (http://mixomics.org/). In addition, a differential ASV abundance analysis based on their negative binomial distribution was performed using the Bioconductor package DESeq2 within R. The *p*-value was adjusted by the Benjamini and Hochberg false discovery rate (FDR) to account for multiple testing. The level of significance was determined at P_adj_ < 0.01 and |Log2Fold Change| ≥ 1.

## 3. Results

### 3.1. Alpha and Beta Diversity

A total of 9.8 M raw reads were obtained from the high-throughput sequencing of the 16S rRNA gene, whereas 3359 ASVs were obtained by performing de novo ASV clustering. Rarefaction was performed on an ASV table rarefied to an equal sampling depth of 4505 sequences/sample.

**Figure 1:**
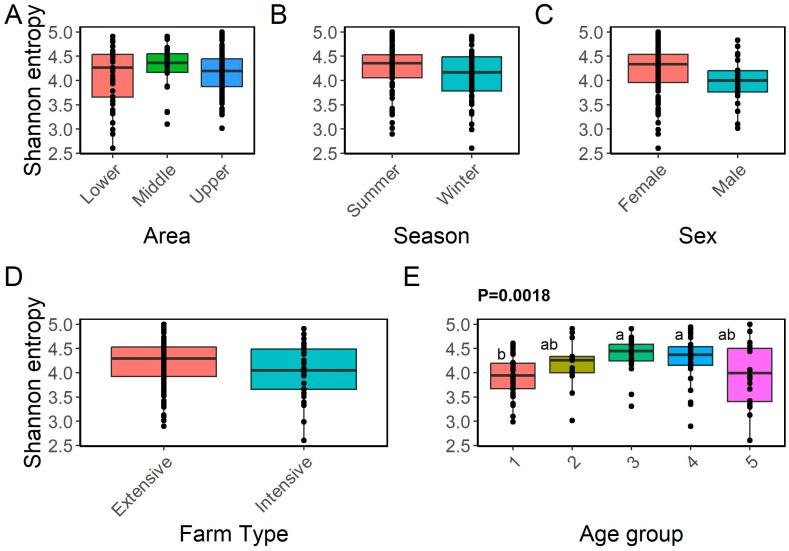
Alpha diversity analysis using the Shannon index as the measure. Boxplots represent variations in the alpha diversity of the microbial communities of local Egyptian cattle breeds per area (**A**), season (**B**), sex (**C**), farm type (**D**), and age group (**E**) (≤2 years, 3–4 years, 5–6 years, 7–8 years, and ≥8 years). ^a,b^ Boxplots with different letters are substantially different from one another (*p* ≤ 0.05).

**Figure 2:**
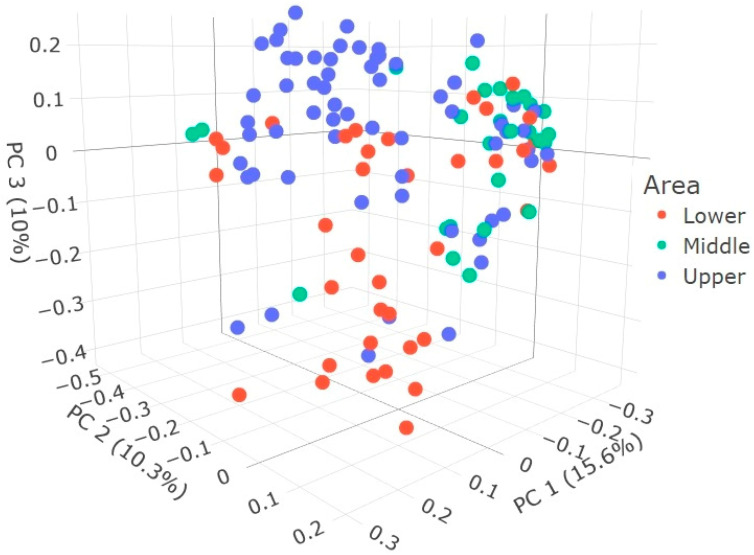
PLSD score plot for beta diversity patterns of microbial communities within fecal samples from Egyptian cattle raised in three different areas (ecosystems). PC1: component 1 (15.6% explained variance), PC2: component 2 (10.3% explained variance), PC3: component 3 (10% explained variance).

The microbial community’s richness and diversity (Figure 1A–E) were represented by the Shannon diversity index, which indicated that the fecal microbiota of the Egyptian cattle was not significantly diverse at the area, season, sex, or farm-type level. Meanwhile, the microbiota data revealed significant differences in richness among age groups (*p* = 0.0018). The PLSD analysis of beta diversity was able to explain about 26% of the variance between different samples using two components. Meanwhile, a third component was able to explain 10% of the variance between different samples, bringing the total explained variance to 36% when using the three components (Figure 2).

### 3.2. Fecal Microbial Community Composition

**Figure 3:**
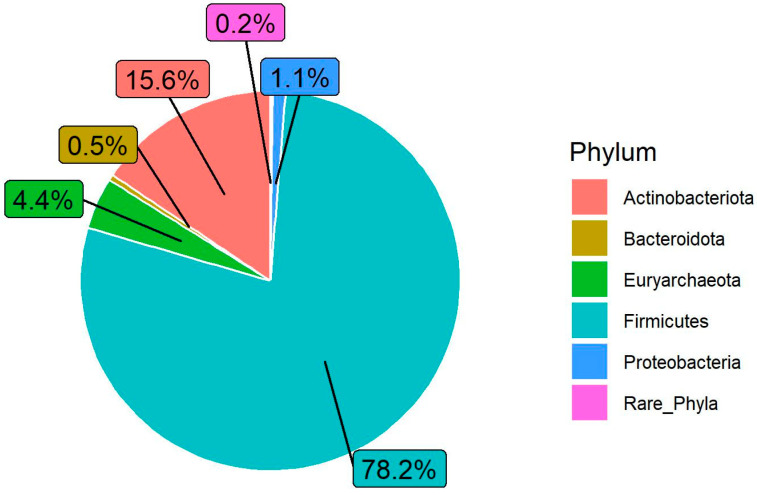
Microbial community composition (at the phylum level) of Egyptian cattle’s fecal microbiota (average for all animals tested in different locations). Phyla with a relative abundance of less than 0.2% are included in the “rare phyla” group.

The fecal microbial community’s composition and structure were analyzed at different taxonomical levels. In general, the taxonomic profiles of the Egyptian cattle’s fecal microbiome (Figure 3) at the phylum level showed a dominance of *Firmicutes* (78.2%) followed by *Actinobacteriota* (15.6%) and *Euryarchaeota* (4.4%) and lower proportion of *Proteobacteria* (1.1%) and *Bacteroidota* (0.5%).

**Figure 4:**
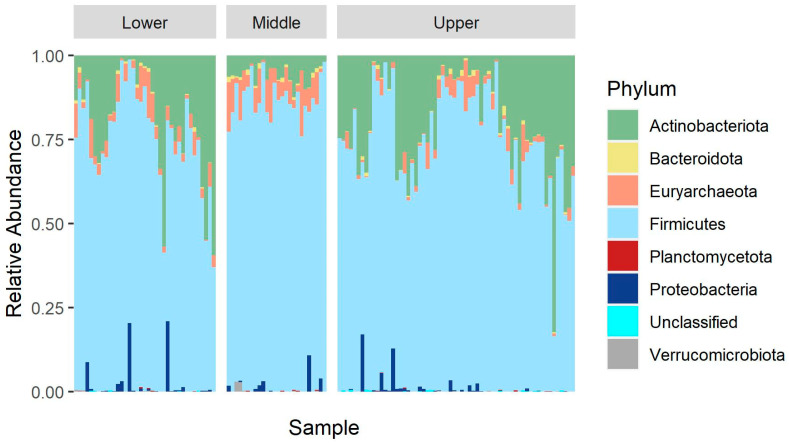
Distribution of the dominant phyla in the fecal microbiota of local cattle breeds from different geographical areas in Egypt.

**Figure 5:**
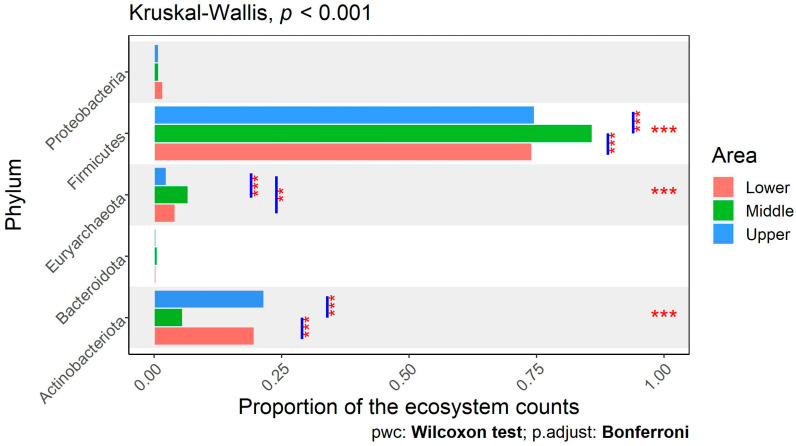
Comparison of the proportions of the dominant phyla in the fecal microbiota of cattle breeds from three different areas of Egypt. Stars represent statistically significant differences in group (area) comparisons within each phylum, using a Kruskal–Wallis test, and pairwise comparisons between the lower, middle, and upper areas of Egypt, using the Wilcoxon test (**, *p* < 0.01; ***, *p* < 0.001).

Analysis of the microbiota data revealed significant differences in abundance (at the phylum, family, and genus level) rather than in richness (*p* > 0.05, alpha diversity for area) among different ecosystems. Figure 4 demonstrates the microbial community’s composition at the phylum level for each tested sample, arranged by different ecosystems. Additionally, Figure 5 indicates the proportion of the different microbial phyla in each ecosystem. Cattle bred in Middle Egypt had a significantly (*p* <0.001) lower relative abundance of *Actinobacteriota* (0.06 ± 0.03) than the ones from Lower (0.20 ± 0.16) and Upper Egypt (0.22 ± 0.15). Meanwhile, *Firmicutes* had a significantly higher relative abundance in cattle from Middle Egypt (0.86 ± 0.06) than the other two regions (0.74 ± 0.14 for Lower and 0.75 ± 0.15 for Upper Egypt) (Figure 5). *Bacteroidota* did not differ significantly between the different areas. However, when calculating the *Firmicutes/Bacteroidota* ratio, there were no significant (*p* > 0.05) differences among areas. Upper Egypt cattle had the lowest relative abundance of *Euryarchaeota*. In the case of *Proteobacteria*, high variation was observed among individuals (samples) in the three ecosystems (Lower, Middle, and Upper Egypt) (Figure 4), but no significant differences were observed in its overall abundance (Figure 5). Meanwhile, the *Verrucomicrobiota* phyla only appeared in Middle Egypt cattle.

**Figure 6:**
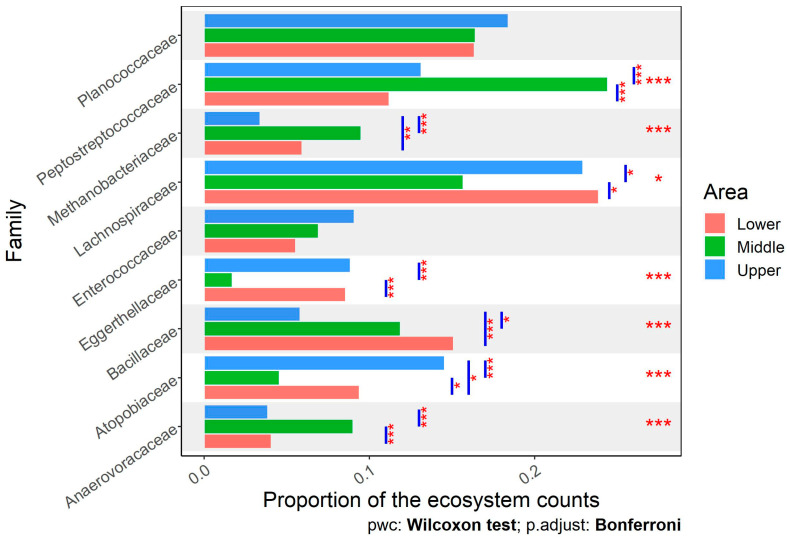
Comparison of the proportions of the dominant families between fecal samples of cattle from different regions of Egypt. Stars depict statistically significant differences in group (area) comparisons within each phylum, using the Kruskal–Wallis test, and pairwise comparisons between the lower, middle, and upper areas of Egypt, using the Wilcoxon test (*, *p* < 0.05; **, *p* < 0.01; ***, *p* < 0.001).

At the family level, Anaerovoracaceae, Atopobiaceae, Bacillaceae, Eggerthellaceae, Lachnospiraceae, Methanobacteriaceae, Peptostreptococcaceae, Enterococcaceae, and Planococoaceae were the most abundant families in the fecal samples of local Egyptian cattle raised in three different ecosystems (Figure 6). Seven out of the nine most abundant families were significantly different among ecosystems. Cattle from Middle Egypt had almost double the proportion of Anaerovoracaceae and Peptostreptococcaceae families compared with cattle from Lower and Upper Egypt. On the other hand, Atopobiaceae and Lachnospiraceae were proportionally lower in cattle from Middle Egypt than those from the other two regions. The greatest proportion of the Methanobacteriaceae family was in cattle from Middle Egypt, followed by Lower Egypt, while it was lowest in Upper Egypt.

**Figure 7:**
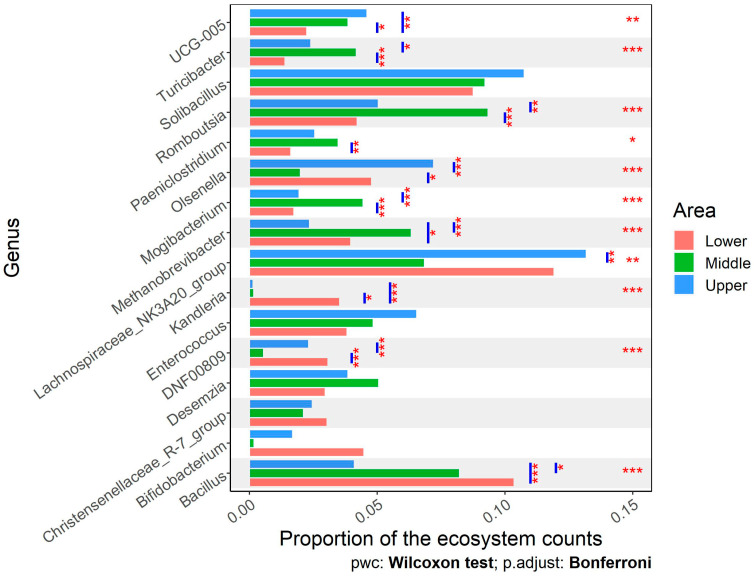
Comparison of the proportions of the dominant genera in the fecal microbiota of cattle bred in different areas of Egypt. Stars depict statistically significant differences in group (area) comparisons within each phylum, using the Kruskal–Wallis test, and pairwise comparisons between the lower, middle, and upper areas of Egypt, using the Wilcoxon test (*, *p* < 0.05; **, *p* < 0.01; ***, *p* < 0.001).

At the genus level, 86.5% of the ASVs were assigned to known genera. Figure 7 demonstrates the most abundant genera, presented as a proportion of each ecosystem’s counts. The *Lachnospiraceae_NK3A20_group* was the most abundant genus in cattle from Upper and Lower Egypt (13.1% and 11.9%, respectively), meanwhile, *Romboutsia* (9.3%) and *Solibacillus* (9.2%) were the most abundant genera in cattle from Middle Egypt. *Bacillus* had a significantly higher abundance in cattle raised in Lower and Middle Egypt than those managed in Upper Egypt. *Methanobrevibacter* had a significantly higher abundance in cattle from Middle Egypt (6.3%) than Lower Egypt (4%) and Upper Egypt (2.3%). The *Kandleria* genus showed a significantly higher abundance in Lower Egypt (3.5%) than Middle (0.16%) and Upper Egypt cattle (0.14%).

**Figure 8:**
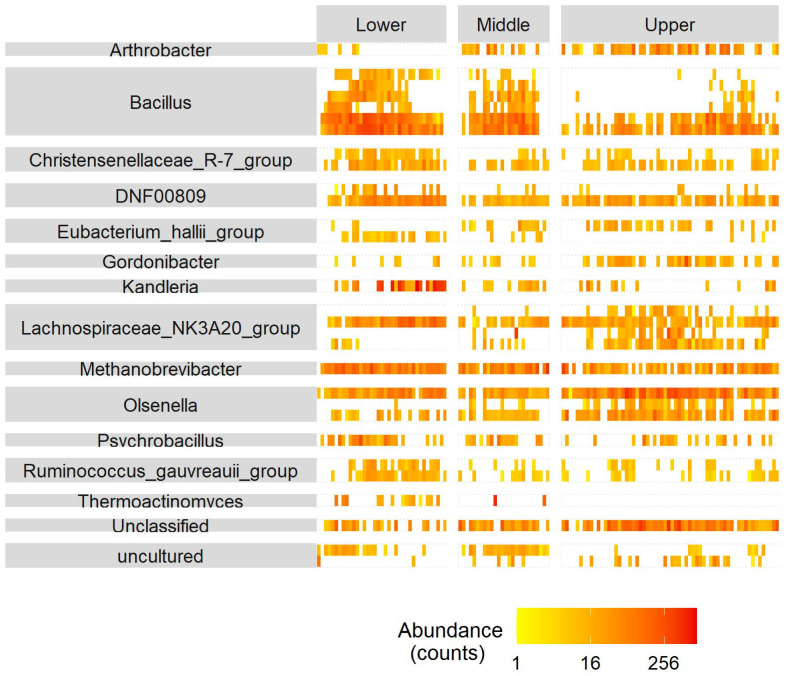
Heatmaps representing the abundance of ASVs across all samples and their genus group. Only ASVs differentially abundant between areas (Lower, Middle, and Upper Egypt) were included in the heatmaps (P_adj_ < 0.01 and |Fold Change| ≥ 1).

To identify specific organisms in the microbiota that were affected by the area where the cattle breeding was managed, a differential abundance analysis was performed for the different regions. There were 30 ASVs that showed significant differences (FDR < 0.01 and −1 > Log2 fold change > 1) in their abundance between different breeding areas (Figure 8). Out of the 30 ASVs, there were 6 belonging to *Bacillus* which were higher in cattle bred in Lower Egypt than in the other two areas. In addition, there were four ASVs belonging to the *Lachnospiraceae_NK3A20_group* which showed a higher abundance in cattle bred in Upper than in Middle and Lower Egypt.

## 4. Discussion

The gut microbiome of cattle affects several physiological processes, including immune system development [28], animal productivity [29], and adaptation [9,10]. This study aimed to gain insights into the adaptation of the microbial communities of local Egyptian cattle raised in different production environments. Several previous studies have confirmed that age is one of the main factors influencing the diversity and composition of microbial communities [30,31]. Consistent with these studies, our results showed that cattle of different ages within the same ecosystem have significantly different fecal microbiota compositions [30,31]. As the effect of age on the microbiome’s diversity and composition is well known and documented [30,31], and since we have confirmed that different age groups are represented and distributed across the three ecosystems, we did not include this factor in our subsequent analyses in order to focus on differences that were due to variations in these ecosystems.

The taxonomic analysis of the 16S rRNA gene amplicon reads identified *Firmicutes* and *Actinobacteriota* as the dominant phyla, which together accounted for over 93% of the total microbial population in Egyptian cattle. Several earlier studies confirmed that *Firmicutes* is typically the most dominant phylum, followed by *Bacteroidota* [11,29,32]. In contrast, our results revealed that *Bacteroidota* represented only 0.5% of the total microbial population. The lower abundance of *Bacteroidota* could be explained by several factors such as diet type [33] and the cattle’s adaptive ability to produce milk and meat under heat stress [11]. Diet type has been shown to significantly affect the fecal microbiota’s composition. For example, the abundance of *Bacteroidota* in the feces of cattle fed a silage/forage diet accounted for only 1.83% of all sequences and was the fourth most dominant phylum; meanwhile, elsewhere, it accounted for 37.39% of all sequences in cattle fed a moderate grain diet [33]. The low *Bacteroidota* abundance observed in this study could be partially explained by the fact that farmers in developing tropical and subtropical countries often rely on feeding their local animals/breeds low-quality protein and energy sources such as low-grade grass [34]. This highlights the role of the adaptation period, which is needed when transitioning to or being supplied with a high-grain diet to develop a balanced gut microbiota [35].

Additionally, the increased abundance of *Firmicutes* in the bovine gut suggests a high efficiency in nutrient utilization under high-temperature environments [32,36]. It has been suggested that regulating the *Firmicutes* population in the bovine gut microbiome can occur through increasing its abundance, which contributes to the adaptation of cattle to maintain their production performance during heat stress [11]. Unexpectedly, the abundance of *Firmicutes* was significantly increased in the fecal microbiota of cattle raised in Middle Egypt compared to those raised in Upper Egypt under hotter temperatures (20.88 to 36.97 °C in Middle Egypt versus 22.37 to 38.89 °C in Upper Egypt during the summer). This can be explained by the high THI recorded in Middle Egypt (max 87.02) compared to Upper Egypt (max 83.09) due to the high humidity in Middle Egypt. In this context, the relatively high abundance of *Firmicutes* in cattle raised in Middle Egypt was accompanied by a low abundance of *Actinobacteriota* compared to those managed in Lower and Upper Egypt. In other studies, the abundance of *Actinobacteriota* was higher in samples from arid regions [37] and in bovine raised under heat stress conditions compared to those in temperate climates [11]. This suggests a difference in the adaptation of the microbial community in cattle raised in Middle Egypt. In general, the high abundance of the *Firmicutes* phyla was explained at the genus level by the high abundance of *Bacillus*, *Lachnospiraceae_NK3A20_group*, *Romboutsia*, and *Solibacillus*, which represent the genera with the highest abundances.

Methane is produced in the rumen of artiodactyl animals by a group of *Archaea* known collectively as methanogens, which belong to the phylum *Euryarcheota*. In our study, the relative abundance of the *Euryarcheota* phylum was significantly different among cattle raised in different ecosystems. Additionally, at the family level, *Methanobacteriaceae* were significantly more abundant in cattle from Middle Egypt, and that high abundance was mainly explained by the high abundance of the *Methanobrevibacter* genus. In this context, ruminal methanogenesis is affected by various factors, including diet composition, host species, and geographical locations, which have also been found to influence the methanogen community’s structure [38]. On the contrary, the abundance of the *Lachnospiraceae_NK3A20_group* genus was significantly lower in Middle Egypt than in Lower and Upper Egypt, while it was the most abundant genus in Lower and Upper Egypt. The genus *Lachnospiraceae_NK3A20 group* is among the ten most abundant bacterial genera in 16S rRNA gene surveys of the rumen microbiota and produces the hydrogen that is converted to methane in the rumen [39]. In this context, it is conceivable that several microbial species could perform the same task due to functional redundancy among them, with various combinations of microorganisms being co-selected based on external conditions. Because of the rumen microbial community structure’s adaptability, ruminant hosts can thrive in a range of different environments [40].

The genus *Bacillus* was previously identified as one of the main microorganisms producing propionate in the rumen and showed a relatively high abundance in animals fed with a high concentrate ration [41]. We found that *Bacillus* had significant differences in its abundance among ecosystems, as it was highly abundant in cattle living in Lower compared to Upper Egypt. This could be explained by animals in Upper Egypt usually being fed on agricultural crops and their residues, which tend to be high in fiber [42]. Meanwhile, the intensive system that is dominant in Lower Egypt provides a higher concentrate/forage ratio.

The abundances of the genera *Romboutsia*, *Paeniclostridium*, and *Turicibacter* were increased in calves who had recovered from diarrhea and have been shown to have positive correlations with serum glucose and phosphorus levels, but negative correlations with serum chloride levels [43]. A similar pattern was previously observed in goats, where the percentage of *Romboutsia*, *Paeniclostridium*, and *Turicibacter* was significantly higher in diarrheic kids compared to healthy goats [44]. Furthermore, when studying the relationship between fecal metabolites and the gut microbiota, *Romboutsia* and *Turicibacter* were negatively correlated with butyric acid profiles and were associated with health disorders in rats [45]. This could be explained by the immunomodulatory effects of butyrate, which binds to GPR43, subsequently activating the production of anti-inflammatory cytokines, such as TGFβ and IL-10, as well as upregulating FOXP3 in Treg cells [46]. Meanwhile, *Paeniclostridium* produces a lethal toxin factor that can bind to the host and affect normal glycosylation reactions [47]. Clearly, *Romboutsia*, *Turicibacter*, and *Paeniclostridium* share common characteristics. First, their abundance increases in cases of diarrhea. Second, they adapt to maintain and even promote conditions that lead to diarrhea. In the current study, cattle from Middle Egypt showed a significantly higher abundance of these three genera (*Romboutsia*, *Paeniclostridium*, and *Turicibacter*), which could indicate their exposure to high disease and stress pressures, consistent with the high THI values in that region.

It has been confirmed that signals derived from the gut microbiota are critical for shaping both innate and adaptive immunity [48]. In this context, the *Christensenellaceae* family is regarded as a potentially beneficial group of bacteria due to its role in regulating the intestinal environment and its links to immunomodulation and health homeostasis [49]. In our study, *Christensenellaceae* did not differ significantly among the three ecosystems; however, they were one of the ten most abundant genera in all ecosystems. This could be attributed to the high adaptability of the local Egyptian cattle breed.

## 5. Conclusions

To our knowledge, this is the first study in Egypt that has established a reference profile of the microbiota diversity of local Egyptian cattle being bred in three different ecosystems. The significant differences in the microbiota composition that were found among cattle developed in different geographical areas (ecosystems) suggest an adaptive response of the animals to their respective/specific environment, with a clear effect of heat stress and feed type. Additional studies in this area are needed to gather more details concerning other phenotypes such as the production and feeding traits of the cattle in the various ecosystems.

## Figures and Tables

**Table 1:** Average ambient temperature (°C), relative humidity (%), and temperature–humidity index (THI) during the hot and cold seasons.

Region	Season	Average Ambient Temperature (°C)		Average Relative Humidity (RH %)	THI	
		Min	Max		Min	Max
Lower	Hot	22.37	38.89	26.96	66.32	83.90
	Cold	7.54	25.12	30.52	50.15	69.41
Middle	Hot	20.88	36.97	49.43	66.27	87.02
	Cold	8.20	21.52	59.52	49.31	67.52
Upper	Hot	21.98	35.14	55.72	68.15	86.04
	Cold	8.05	26.55	60.52	58.86	74.93
Overall	Hot	21.74	37.0	44.04	66.91	85.66
	Cold	10.26	24.31	50.18	52.87	70.62

## Data Availability

The raw data supporting the conclusions of this article will be made available by the corresponding author on request.
